# Geostatistical models using remotely‐sensed data predict savanna tsetse decline across the interface between protected and unprotected areas in Serengeti, Tanzania

**DOI:** 10.1111/1365-2664.13091

**Published:** 2018-02-13

**Authors:** Jennifer S. Lord, Stephen J. Torr, Harriet K. Auty, Paddy M. Brock, Mechtilda Byamungu, John W. Hargrove, Liam J. Morrison, Furaha Mramba, Glyn A. Vale, Michelle C. Stanton

**Affiliations:** ^1^ Department of Vector Biology Liverpool School of Tropical Medicine Liverpool UK; ^2^ Epidemiology Research Unit SRUC Inverness UK; ^3^ Institute of Biodiversity, Animal Health and Comparative Medicine College of Medical, Veterinary and Life Sciences University of Glasgow Glasgow UK; ^4^ Vector and Vector‐Borne Diseases Research Institute Tanga Tanzania; ^5^ SACEMA University of Stellenbosch Stellenbosch South Africa; ^6^ Roslin Institute Royal (Dick) School of Veterinary Studies University of Edinburgh Midlothian UK; ^7^ Natural Resources Institute University of Greenwich Chatham UK

**Keywords:** disease, geostatistical models, *Glossina*, pathogens, protected areas, remote‐sensing, surveillance, trypanosomiasis, tsetse, vector control

## Abstract

1. Monitoring abundance is essential for vector management, but it is often only possible in a fraction of managed areas. For vector control programmes, sampling to estimate abundance is usually carried out at a local‐scale (10s km^2^), while interventions often extend across 100s km^2^. Geostatistical models have been used to interpolate between points where data are available, but this still requires costly sampling across the entire area of interest. Instead, we used geostatistical models to predict local‐scale spatial variation in the abundance of tsetse—vectors of human and animal African trypanosomes—beyond the spatial extent of data to which models were fitted, in Serengeti, Tanzania.
2. We sampled *Glossina swynnertoni* and *Glossina pallidipes* >10 km inside the Serengeti National Park (SNP) and along four transects extending into areas where humans and livestock live. We fitted geostatistical models to data >10 km inside the SNP to produce maps of abundance for the entire region, including unprotected areas.
3. Inside the SNP, the mean number of *G. pallidipes* caught per trap per day in dense woodland was 166 (± 24 *SE*), compared to 3 (±1) in grassland. *Glossina swynnertoni* was more homogenous with respective means of 15 (±3) and 15 (±8). In general, models predicted a decline in abundance from protected to unprotected areas, related to anthropogenic changes to vegetation, which was confirmed during field survey.
4. *Synthesis and applications*. Our approach allows vector control managers to identify sites predicted to have relatively high tsetse abundance, and therefore to design and implement improved surveillance strategies. In East and Southern Africa, trypanosomiasis is associated with wilderness areas. Our study identified pockets of vegetation which could sustain tsetse populations in farming areas outside the Serengeti National Park. Our method will assist countries in identifying, monitoring and, if necessary, controlling tsetse in trypanosomiasis foci. This has specific application to tsetse, but the approach could also be developed for vectors of other pathogens.

## Abstract 2

Our approach allows vector control managers to identify sites predicted to have relatively high tsetse abundance, and therefore to design and implement improved surveillance strategies. In East and Southern Africa, trypanosomiasis is associated with wilderness areas. Our study identified pockets of vegetation which could sustain tsetse populations in farming areas outside the Serengeti National Park. Our method will assist countries in identifying, monitoring and, if necessary, controlling tsetse in trypanosomiasis foci. This has specific application to tsetse, but the approach could also be developed for vectors of other pathogens.

## 1 INTRODUCTION

In sub‐Saharan Africa, the edges of protected areas, including national parks and game reserves, have experienced human population growth twice that of other rural areas (Wittemyer et al., 2008). These regions present a complex transmission context for zoonotic diseases, including Rhodesian human African trypanosomiasis (r‐HAT; Auty, Morrison, Torr, & Lord, 2016; Hassell, Begon, Ward, & Fèvre, 2016). In these areas, *Trypanosoma brucei rhodesiense*—the causative agent of r‐HAT—circulates in wildlife and livestock, alongside the trypanosome species which cause animal African trypanosomiasis (AAT). A better understanding of trypanosome transmission dynamics and approaches to improve control are current priorities for these regions (Auty et al., 2016; Diall et al., 2017).

Tsetse control is the only method available for reducing r‐HAT within and around the edges of protected areas. Savanna tsetse—the Morsitans group of *Glossina*—are the primary vectors of the trypanosomes which cause r‐HAT and AAT inside and adjacent to protected areas in east and southern Africa (Gondwe et al., 2009; Mweempwa et al., 2015). For livestock‐owning communities, use of insecticide‐treated cattle is the most cost‐effective method of tsetse control but it requires sufficient densities of cattle (Shaw et al., 2015). Where cattle are sparse, insecticide‐impregnated targets baited with attractants can be used. Application of these vector control methods over large and remote protected areas is not feasible, but control can be targeted in interface areas to reduce human and livestock exposure. In order to concentrate resources for optimal cost‐effective control requires information on the distribution and abundance of tsetse at a local level—10s km^2^, but district/country‐level maps are often only available (Albert, Wardrop, Atkinson, Torr, & Welburn, 2015; Dicko et al., 2014; Hendrickx et al., 1999; Wint & Rogers, 2000). Although atlases of HAT and AAT exist (Cecchi et al., 2014; Simarro et al., 2010), for Tanzania, local‐scale and contemporary knowledge of tsetse distribution and abundance is lacking.

Extensive grid‐based sampling of tsetse using traps to estimate abundance coupled with vegetation mapping using remotely‐sensed data has previously been applied to inform tsetse control (Albert et al., 2015; Dicko et al., 2014; Hendrickx et al., 1999). However, such sampling is logistically intensive and expensive. This is particularly so within or at the edges of protected areas which are often remote and difficult to access. The ability to predict where vegetation is suitable for tsetse, using remotely‐sensed data, would be valuable for informing surveillance and control (Kalluri, Gilruth, Rogers, & Szczur, 2007).

Remotely‐sensed data can be included in geostatistical models to identify areas where vegetation may be suitable for tsetse (Albert et al., 2015; Bouyer et al., 2010; Dicko et al., 2014; Ducheyne et al., 2009; Kitron et al., 1996; Mweempwa et al., 2015). However, few studies have linked tsetse abundance, habitat and remotely‐sensed variables on a local scale (Kitron et al., 1996). Moreover, local‐scale statistical models for tsetse have not been tested for their ability to predict abundance in regions other than those for which the original model was produced (Albert et al., 2015; Bouyer et al., 2010; Dicko et al., 2014; Ducheyne et al., 2009; Kitron et al., 1996; Mweempwa et al., 2015).

In regions >10 km inside protected areas, the drivers of tsetse population dynamics are limited to be natural variation in vegetation and wildlife densities associated with vegetation (Allsopp, Baldry, & Rodrigues, 1972). Rather than interpolation (Albert et al., 2015; Dicko et al., 2014; Hendrickx et al., 1999), comparing extrapolated predictions from models fitted to data inside protected areas with data from across the interface would be a way of testing the robustness of relationships between remotely‐sensed data as indicators of habitat and tsetse abundance. It may also allow insight into the drivers of tsetse distribution at the interface between protected and unprotected areas (Miller, Turner, Smithwick, Dent, & Stanley, 2004).

We quantified mean daily numbers of tsetse caught in traps as a function of vegetation type, for areas >10 km inside the Serengeti National Park (SNP), Tanzania. We then used geostatistical models based on remotely‐sensed data fitted to these tsetse catches to predict how abundance varies spatially in regions across the interface between protected and unprotected areas. We tested model predictions with new field data from the interface.

## 2 MATERIALS AND METHODS

### 2.1 Study area

**Figure 1:**
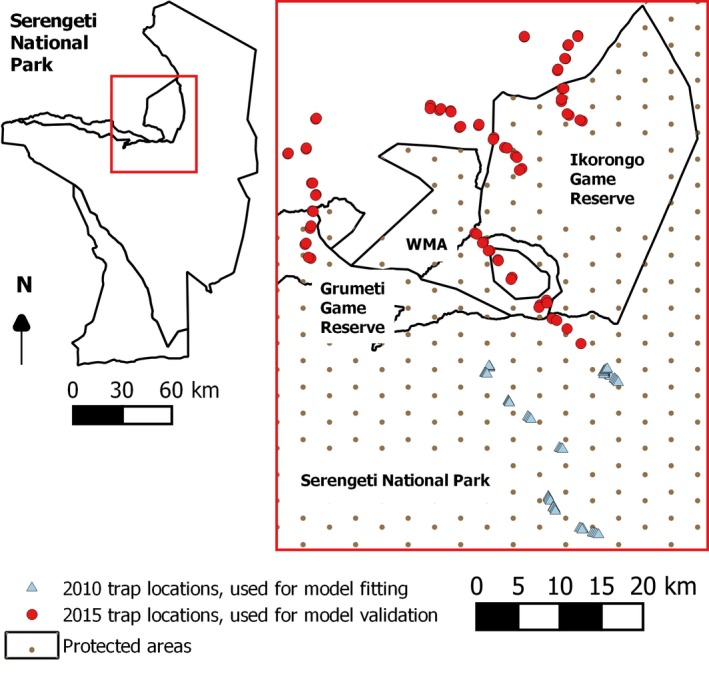
Study area and Nzi trap locations. Triangles: 2010 Nzi trap sampling sites >10 km inside the Serengeti National Park. Circles: 2015 Nzi trap sampling sites across the interface between protected and unprotected areas. WMA, wildlife management area [Colour figure can be viewed at wileyonlinelibrary.com]

The protected areas in the study, comprising approximately 3,000 km^2^ (Figure 1) support a gradient of habitats from dense woodland to grassland (Reed, Anderson, Dempewolf, Metzger, & Serneels, 2009). To the northwest of the SNP, communities practice livestock keeping and mixed crop‐livestock farming. Some unprotected areas here however still support natural vegetation (Estes, Kuemmerle, Kushnir, Radeloff, & Shugart, 2012). The northern region of Tanzania, including the SNP, has been known as an r‐HAT focus since the 1920s (Davey, 1924; Swynnerton, 1923). More recently, human infective *T. brucei rhodesiense* has been identified by PCR in cattle and wildlife in the study area (Auty, 2009; Kaare et al., 2007). Although r‐HAT cases in residents are likely underreported (Odiit et al., 2005), 30 cases in travellers to the region were reported between 1990 and 2007 (Auty, 2009). The SNP supports three species of tsetse: *Glossina swynnertoni*,* Glossina pallidipes* and *Glossina brevipalpis*—although the last species is present in smaller numbers than Morsitans group species (Auty et al., 2012).

### 2.2 Quantifying tsetse abundance

Surveys were carried out in February 2010 >10 km inside the SNP and in February 2015 across the interface between protected and unprotected areas (Figure 1). Sampling was carried out in February, prior to the rains to minimise water damage to traps and because tsetse abundance in Tanzania has previously been found to be highest between February and June (Glasgow & Welch, 1962). Tsetse were sampled using Nzi traps (Mihok, 2002) baited with a blend of acetone (100 mg/hr), 1‐octen‐3‐ol (0.5 mg/hr), 4‐methylphenol (1 mg/hr) and 3‐*n*‐propylphenol (0.1 mg/hr; Torr, Hall, Phelps, & Vale, 1997). Trap locations were recorded using a GPS. Catches from traps were collected daily for three consecutive days and the numbers of tsetse of each sex and species were recorded. We use the term “abundance” to describe mean numbers of tsetse caught per trap per day.

In February 2010, traps were deployed in each of four vegetation types, categorised, using an existing vegetation map (Hopcraft, 2008) and ground‐truthing, as grassland (<2% tree cover), savanna (2%–20% tree cover), open woodland (20%–50% tree cover) and dense woodland (50%–100% tree cover). Traps were deployed along three transects (Figure 1) extending from *c*. 10 to *c*. 30 km inside the SNP, based on accessibility from roads. Eighteen traps were placed in dense woodland, 14 in grassland, 14 in open woodland and 14 in savanna. Traps in each vegetation type were at least 50 m apart in dense/open woodland or 100 m apart in grassland/savanna, because traps are more visible to tsetse in open areas.

In February 2015, four transects of Nzi traps were set from 5 km inside, up to 10 km outside the protected area boundary (Figure 1). Transects were selected based on their accessibility from roads. Along each transect, we set pairs of odour‐baited Nzi traps at approximately 1.5‐km intervals irrespective of vegetation type. The Euclidean distance of traps from the protected area boundary was estimated.

We assume that our trapping did not affect the tsetse population. An odour‐baited insecticide‐treated target, which will catch similar numbers of tsetse as an odour‐baited trap, kills *c*. 1% of tsetse per day within a km^2^ (Vale, Hargrove, Cockbill, & Phelps, 1986). To have an impact on a tsetse population requires >3% of the population within a km^2^ to be captured or killed per day and this has to be applied for months to impact the population (Hargrove, 1988).

### 2.3 Remotely‐sensed variables

Numbers of *G. pallidipes* caught in traps have previously correlated inversely with Landsat shortwave infrared values—an indicator of moisture (Barsi, Lee, Kvaran, Markham, & Pedelty, 2014; Kitron et al., 1996). Normalized Difference Vegetation Index (NDVI) values above 0.39 have previously been used as an indicator of vegetation suitable for tsetse (Lin, DeVisser, & Messina, 2015; Moore & Messina, 2010), based on the observation that mortality rates decrease as NDVI increases (Rogers & Randolph, 1991). The NDVI is a measure of the density of plant matter—using the near‐infrared and visible red wavelengths. We also included elevation and land surface temperature (LST), given the importance of temperature to tsetse mortality rates (Hargrove, 2004). For estimates of LST, the Landsat 8 Thermal Infrared Band 10 image was converted to at‐satellite brightness temperature. We then used NDVI to estimate emissivity and calculate LST as previously described (Sobrino, Jiménez‐Muñoz, & Paolini, 2004). For elevation, we used the ASTER Global Digital Elevation Model (GDEM), which is a product of NASA and METI and has a resolution of 30 × 30 m.

Three Landsat 8 images (30 × 30 m resolution) with less than 10% cloud cover—Path/Row 169/061, 169/062 and 170/061—and the GDEM were acquired from Earth Explorer (https://earthexplorer.usgs.gov/) from 13 February 2015 and 20 February 2015. The 2015 images had lower cloud cover than that available from 30 January 2010, and both sets of images appeared sufficiently similar to use the 2015 set for both time points. Histogram matching, conversion to near‐surface reflectance, calculation of NDVI and application of a cloud mask was done in r (R Core Team, 2014).

### 2.4 Data analysis

Only 56 *G. brevipalpis* were caught throughout our studies and numbers were too low for analysis. *Glossina pallidipes* and *G. swynnertoni* count data were overdispersed. To compensate for this, daily catches (*y*) from 3 days for each trap were transformed to log_10_(*y *+ 1) before calculation of mean numbers per trap per day and *SE*.

For each species, we first explored the variation in numbers per trap per day, >10 km inside the SNP during February 2010, between vegetation types. To determine the significance of the difference in log‐transformed abundance observed between vegetation types for each species, we used ANOVA followed by the Tukey Honest Significant Difference test (Tukey HSD). We then fitted a Bayesian linear geostatistical model (Brown, 2015; Diggle, Tawn, & Moyeed, 1998) only to the February 2010 data from >10 km inside the SNP. The function glgm within geostatsp (Brown, 2015) was used as it provided a user‐friendly method for fitting and comparing models within a Bayesian framework. The glgm function implements integrated nested Laplace approximation (Rue, Martino, & Nicolas, 2009) for fitting and prediction and outputs cross‐validation measures for model comparison.

Allowing for savanna tsetse daily dispersal rates (Hargrove, 1981), a buffer of 500 m around each trap was used to calculate the average LST, Landsat Band 7, NDVI and elevation. We used a systematic model fitting approach focused on the predictive ability of the model, rather than identifying a causal relationship between the environmental variables and the trap data. Separate linear regression models were first fitted using each of the four individual environmental variables. Model fits, initially without a spatially explicit error term, to the *G. pallidipes* and *G. swynnertoni* data, were compared using the negative of the sum of the log conditional predictive ordinates (log‐CPO score; Held, Schrödle, & Rue, 2010). Conditional predictive ordinates indicate the ability of a model to predict the value of a data point omitted from the fitting step. Starting with the univariate model with the lowest log‐CPO score, each of the remaining variables was added to the model one at a time. The log‐CPO was recalculated with each additional variable, and the combination with the lowest log‐CPO was determined to be the most parsimonious.

Rasters of environmental variables remaining in the most parsimonious model were resampled to a resolution of 500 × 500 m, considering tsetse daily dispersal rates and to reduce computing time, covering an area including both 2010 transects inside the SNP and 2015 transects across the interface. We then compared the fit, to the 2010 data, of a geostatistical model including a spatially structured error term to a model including an unstructured error term by comparing log‐CPO scores. Normally distributed errors were assumed, and prior 95% intervals for the practical range were 500 and 5,000 m.

To test the ability of our models to predict tsetse abundance, predictive maps were simultaneously produced. Log_10_(*y* + 1) transformed geostatistical model predictions for new locations sampled during 2015 across the interface were then compared with log_10_(*y* + 1) transformed observed values and residuals between predicted and observed counts calculated.

All data and r scripts required to produce the figures are available online (Lord et al., 2018).

## 3 RESULTS

### 3.1 Tsetse abundance >10 km inside the SNP

**Figure 2:**
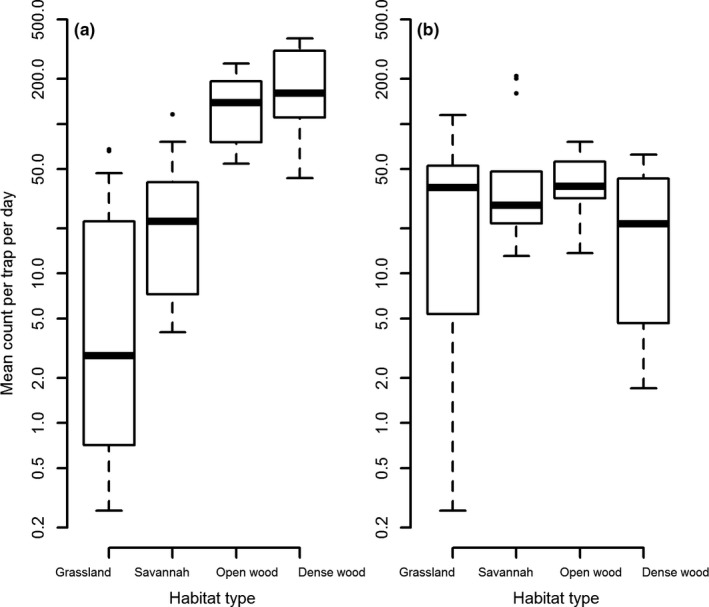
Number of tsetse caught per trap per day by habitat type inside the Serengeti National Park, February 2010. *Y*‐axes on a log scale. (a) *Glossina pallidipes* was significantly lower in grassland and savanna than in open and dense woodland and was lower in grassland compared with savanna (ANOVA *F* 38.46, *p *< .001, Tukey HSD *p* < .001 for each pairing with either grassland or savanna, *p* = .02 for grassland‐savanna); (b) *Glossina swynnertoni*—no significant difference between habitat types (*p* > .05)

During 2010 surveys >10 km inside the SNP, mean numbers of *G. pallidipes* per trap per day were 123 (±24) in open woodland, and 166 (±17) in dense woodland (Figure 2a). This was *c*. 30 times greater than that observed in grassland habitat and six times greater than in savanna habitat (Figure 2a). The mean number of *G. swynnertoni* caught per trap per day was more homogenous across vegetation types (Figure 2b).

For *G. pallidipes*, the model, using remotely‐sensed data, with the lowest log‐CPO score included all the environmental variables (Table 1). Although the distribution of *G. swynnertoni* appeared homogenous between habitat types (Figure 2b), the model including NDVI, elevation and Band 7 had better predictive ability than other models (Table 1). The mean NDVI between grassland and savanna sites was similar—0.40 (±0.01) in grassland and 0.38 (±0.01) in savanna which may indicate why similar numbers of *G. swynnertoni* were caught between these two habitats.

**Table 1:** Negative of the sum of the log conditional predictive ordinates (log‐CPO score) for linear models using different combinations of remotely‐sensed environmental variables. Variables included Landsat 8 Band 7, Normalized Difference Vegetation Index (NDVI), land surface temperature (LST) and elevation. Lower scores indicate better predictive ability

Remotely‐sensed environmental variables included in model	Log‐CPO score
*Glossina pallidipes*	
Band 7	48.9
NDVI	59.6
LST	57.8
Elevation	59.8
Band 7, elevation	47.8
Band 7, elevation, NDVI	47.1
Band 7, elevation, LST	47.8
Band 7, elevation, NDVI, LST	32.7
*Glossina swynnertoni*	
Band 7	41.1
NDVI	38.0
LST	42.0
Elevation	41.1
NDVI, elevation	29.3
NDVI, elevation, LST	27.3
NDVI, elevation, Band 7	24.2
NDVI, elevation, Band 7, LST	27.3

Models including a spatially structured error term provided better fits to the data for both *G. pallidipes* (log‐CPO score 25.5) and *G. swynnertoni* (log‐CPO score 8.4), than models including an unstructured error term (log‐CPO score 37.5 and 23.4). Posterior estimates of model coefficients for the most parsimonious models including a spatially structured error term are provided in Table 2. The distance beyond which abundance was no longer correlated was 3,323 m for *G. pallidipes* and 2,477 m for *G. swynnertoni*. For both species, the amount by which abundance declines as elevation increases was estimated to be equivalent. However, increases in Band 7—indicating lower land surface moisture—was related to a greater decline in *G. pallidipes* abundance than *G. swynnertoni* abundance.

**Table 2:** Posterior estimates of geostatistical model coefficients. Based on fits to log(*y* + 1) transformed data

	*M*	*SD*	0.025 quantile	0.5 quantile	0.975 quantile
*Glossina pallidipes*					
Intercept	12.42	5.180	0.674	12.90	21.34
NDVI	−7.633	4.147	−14.78	−8.023	1.605
Elevation	−0.003	0.002	−0.007	−0.003	0.002
Band 7	−43.10	10.97	−62.87	−43.79	−19.41
LST	0.074	0.096	−0.124	0.076	0.259
Range	3,323	1,814	1,118	2,900	7,985
*Glossina swynnertoni*					
Intercept	13.09	3.035	6.862	13.15	18.98
NDVI	−10.30	2.723	−15.63	−10.33	−4.793
Elevation	−0.003	0.002	−0.006	−0.004	−0.0004
Band 7	−17.65	6.718	−30.78	−17.71	−4.144
Range	2,477	1,199	951	2,214	5,531

NDVI, Normalized Difference Vegetation Index; Band 7, Landsat 8 Band 7; LST, land surface temperature.

**Figure 3:**
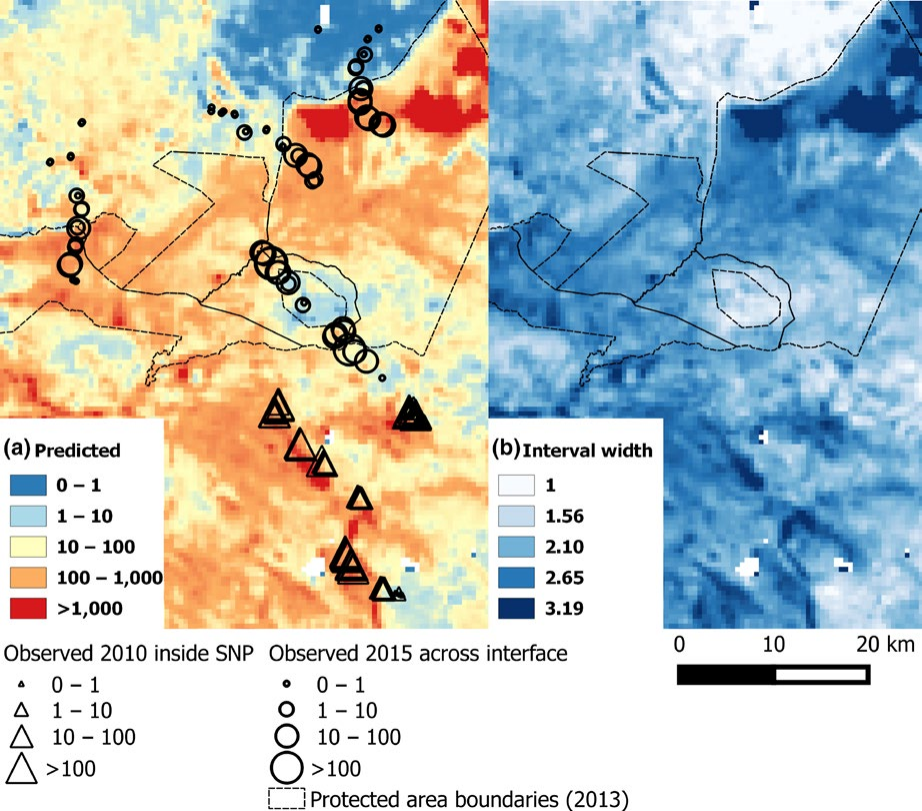
Predictive map of *Glossina pallidipes* abundance based on geostatistical models fitted to 2010 data >10 km inside the Serengeti National Park (triangles). (a) Bayesian posterior mean predicted values, circles—abundance observed during 2015 across the protected area boundary and (b) Bayesian credible interval width (log_10_)—larger values indicating greater model uncertainty in predicted values. See Figure 1 for details on locations of protected areas [Colour figure can be viewed at wileyonlinelibrary.com]

**Figure 4:**
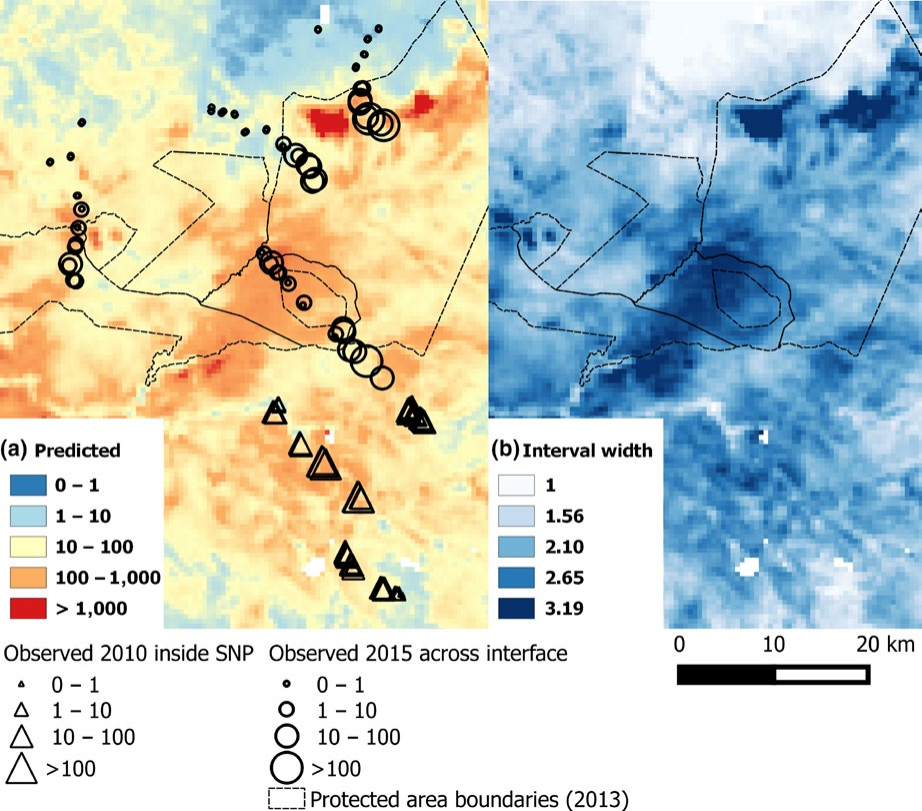
Predictive map of *Glossina swynnertoni* abundance based on geostatistical models fitted to 2010 data >10 km inside the Serengeti National Park (triangles). (a) Bayesian posterior mean predicted values; and (b) Bayesian credible interval width (log_10_)—higher values indicating greater model uncertainty in predicted values. See Figure 1 for details on locations of protected areas [Colour figure can be viewed at wileyonlinelibrary.com]

For both tsetse species, predicted abundance for the sites surveyed in 2010 were overestimates for lower observed values and underestimates for higher observed values (Figure S1). This is likely because the relationships between log‐transformed abundance and remotely‐sensed variables were not exactly linear. However, for both species, maps produced by geostatistical model fits show that most areas predicted to have an abundance >100 were inside the protected area (Figures 3a and 4a). In addition, to the north of Ikorongo Game Reserve, there was a clear change in predicted abundance along the protected area boundary. Inside the protected area, the predicted abundance was >100, but within 5 km into unprotected areas, the predicted abundance was <10. Some unprotected areas to the north of the Grumeti Game Reserve had predicted values of >100 (Figure 3a). *Glossina swynnertoni* was also predicted to be present with abundance >100 in the unprotected areas along the SNP transect (Figures 1 and 4a).

### 3.2 Tsetse abundance at the interface between protected and unprotected areas

**Figure 5:**
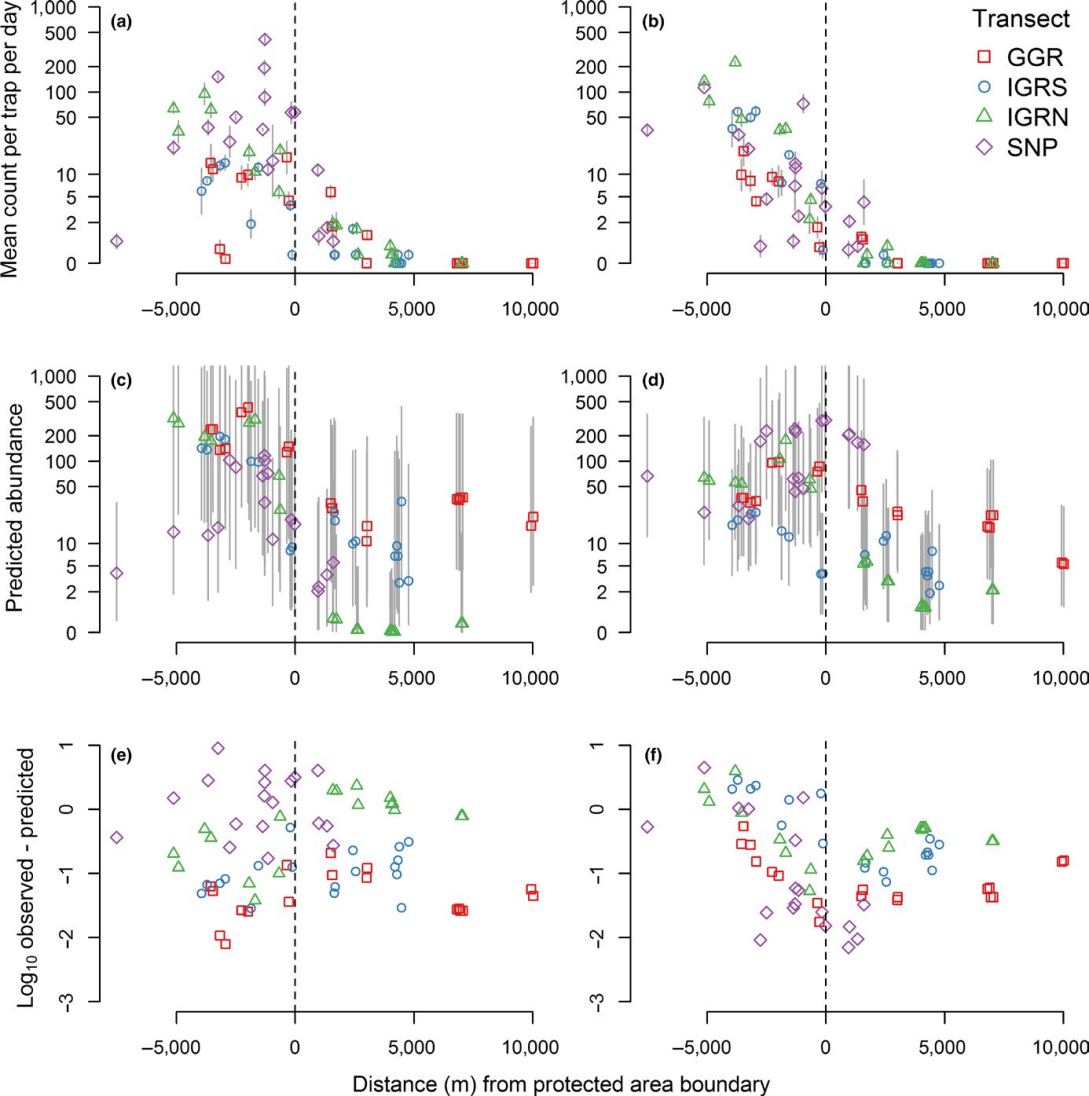
Observed (a, b) and predicted (c, d) tsetse abundance and model residuals (observed—predicted) (e, f) at the interface between protected and unprotected areas. (a) *Glossina pallidipes* observed; (b) *Glossina swynnertoni* observed; (c) *G. pallidipes* predicted; (d) *G. swynnertoni* predicted; (e) *G. pallidipes* residuals; and (f) *G. swynnertoni* residuals. Geostatistical models fitted to 2010 data from >10 km inside the Serengeti National Park. Grey lines in (a) and (b)—*SE*, grey lines in (c) and (d)—95% credible intervals. Negative distance values on the *x*‐axis indicate locations inside the protected area. GGR, Grumeti Game Reserve; IGRS, Ikorongo Game Reserve South; IGRN, Ikorongo Game Reserve North; SNP, Serengeti National Park. For map of trap locations see Figure1 [Colour figure can be viewed at wileyonlinelibrary.com]

Mean values of the environmental variables were similar between 2010 trap sites inside the SNP and the 2015 trap sites across the interface (Table S1). Therefore, the two areas were assumed comparable with respect to the remotely‐sensed variables. At the interface during 2015, on all transects, the abundance of *G. swynnertoni* and *G. pallidipes* declined to zero by 5 km outside the boundary of the protected area in February 2015 (Figure 5a,b). In general, the geostatistical models of abundance also predicted a decline from inside to outside protected areas (Figure 5c,d). Indeed, the protected area boundary to the north of Ikorongo Game Reserve is quite clearly depicted in the predictive map for *G. pallidipes* and similarly so for *G. swynnertoni* (Figures 3a and 4a), even though the models were only fitted to data >10 km inside the SNP. Although a general decline was predicted, model predictions of abundance were over estimates for approximately 80% of trap sites (*G. swynnertoni*—59/72, *G. pallidipes*—55/72; Figure 5e,f). The only locations predicted to have abundance >10, more than 5 km into unprotected areas, were along the Grumeti Game Reserve transect (Figure 5c,d). Field observations confirmed that along this transect there was woody vegetation that may have been sufficient to support tsetse (Figure S2). However, beyond 5 km from the protected area boundary, no tsetse were caught along any of the transects during 2015 surveys. Sites near the boundary where *G. pallidipes* abundance was on average over 50 per trap per day were all associated with riparian vegetation.

## 4 DISCUSSION

Geostatistical models using remotely‐sensed data have potential to identify sites of relatively high vector abundance, for surveillance and control, beyond the spatial extent of initial sampling. We have tested this approach in the case of tsetse flies, by fitting models to the abundance of flies >10 km inside the SNP and testing model predictions across the interface between protected and unprotected areas. In general, the models fitted to data >10 km inside the SNP, predicted a decline in tsetse abundance across the interface. A decline was confirmed by field sampling.

Similar declines in *G. morsitans* have been noted in Zambia and Malawi (Ducheyne et al., 2009; Gondwe et al., 2009; Mweempwa et al., 2015). Our results indicate that loss of woody vegetation explains, in part, such a decline in northern Serengeti, Tanzania. However, our models highlight there may be regions outside protected areas where vegetation is still sufficient to support tsetse populations. Our approach could be used to assist in identifying these remaining regions on a local scale, so that control or monitoring can be targeted effectively.

In our study, sites near the boundary where *G. pallidipes* abundance was on average >50 per trap per day were all associated with riparian woody vegetation. Rivers running from inside to outside protected areas may be where this species could encounter humans and livestock. Control of *G. pallidipes* in this region using insecticide‐treated targets should therefore focus on these riparian habitats.

While *G. pallidipes* was found in higher numbers in dense woodland inside the SNP, the abundance of *G. swynnertoni* did not differ significantly between habitat types. This may be due to the actual density of *G. swynnertoni* being similar between habitat types, or it may be due to trap bias. Indeed, odour‐baited stationary traps are biased towards host‐seeking flies (Hargrove & Packer, 1993). However, the degree of bias is likely different for *G. pallidipes* compared with *G. swynnertoni*. *Glossina pallidipes* is more available to stationary hosts and odour‐baited traps than *G. morsitans* and the latter is more attracted to mobile visual baits relative to stationary ones (Vale, 1974). The resting sites of both species are associated with woody vegetation (Chadwick, 1963; Pilson & Leggate, 1962). If the majority of *G. swynnertoni* and *G. pallidipes* rest in woody vegetation, but a smaller proportion of resting *G. swynnertoni* are stimulated by stationary odour‐baited traps, then we may overestimate the relative importance of *G. pallidipes*.

Although the abundance of *G. swynnertoni* was more homogenous between vegetation types than *G. pallidipes*, models still predicted a decline in numbers from protected to unprotected areas. Remotely‐sensed variables may not correlate directly with habitat type and may reflect other vegetation characteristics important to tsetse. For example, long and short grassland were both grouped as grassland but may have different NDVI values. The similar abundance of *G. swynnertoni* in grassland and savanna habitats may also be due, in part, to the context within which grassland sampling sites were situated with respect to woodland, which we did not account for.

It may be anticipated that regions adjacent to protected areas are subject to relatively high numbers of tsetse dispersing from inside protected areas. However, numbers were already greatly reduced—to 5% of the maximum count in our study space—at the boundary. Our models explained, in part, this observed decline in tsetse abundance, but predicted tsetse to be present in numbers >100 in some unprotected areas. The presence of suitable vegetation at these locations was confirmed by field observations. Even in these regions, abundance was usually <10 at distances 1 km from the boundary, and catches were zero at >5 km from the boundary.

Models generally overestimated abundance at the interface. Overestimates and the predicted more gradual decline across the interface may be expected as the geostatistical models do not account for context. This may also be due, in part, to factors affecting tsetse population dynamics in different ways between 2010 and 2015. A potential limitation to the study is that there was a lack of contemporary Landsat imagery corresponding to the area surveyed in 2010 situated >10 km inside the SNP. The use of 2015 Landsat data to represent conditions in 2010 may therefore explain the model overpredictions in tsetse abundance observed across the interface in 2015, as seasonal conditions in the preceding months may have differed in 2010 in comparison to 2015. However, as the land within the SNP is protected, we expect these changes to be relatively minimal, and unlikely to have a large impact on tsetse habitat. This is supported by the results of the model both with respect to the relationships observed between 2015 Landsat data and 2010 observed relative abundance and in the validation of the model predictions.

The relationships between vector abundance and remotely‐sensed environmental variables are indirect and likely complex, which are not reflected in our linear models. This likely explains why model uncertainty increased with model predicted values. However, the goal of the study was not to predict exact numbers of tsetse, but to find general patterns and establish relative differences between areas that can help to guide surveillance efforts.

Vector‐borne disease control programmes often include reducing the life span of the vectors (Rozendaal, 1997; WHO Expert Committee on Control and Surveillance of Human African Trypanosomiasis, 2013; World Health Organization Global Malaria Programme, 2007). Local spatial variation in vector population dynamics influences the efficiency and success of that strategy (Lambrechts, Knox, Wong, Liebman, &, 2009; Ostfeld, Glass, & Keesing, 2005). Knowledge of local variation in vector abundance is therefore essential in planning the control operation, particularly when a disease is close to elimination or incidence is focal. Vector surveillance at scales relevant to control programmes, but sufficient to quantify local‐scale variation, is however difficult due to logistics, limited resources and costs of intensive sampling (Alimi et al., 2015). The ability to correlate vector abundance with remotely‐sensed data can help direct limited resources (Kalluri et al., 2007). However, the majority use spatial interpolation—predictions made within the same area as the measured data—rather than extrapolation. Extrapolation and validation of predictions within surveillance efforts would require less intensive sampling than grid‐based sampling spanning an entire area planned for control.

We used contemporary open‐source remote‐sensing data. Our approach did not require extensive ground‐based information. The method may therefore be suitable for providing initial local‐scale predictions over 100s km^2^. In addition, given rapid land‐use change occurring at the edges of protected areas (Wittemyer et al., 2008), using models based on remotely‐sensed data which is updated monthly is advantageous.

The resulting models will help managers of vector control programmes to select sites, predicted to have relatively high vector abundance, for surveillance prior to targeting control efforts. This will reduce the chances that suitable sites are missed. Models should then be tested by selecting areas predicted to support relatively high vs. low abundance and these sites subject to tsetse surveillance. This will help guide surveillance to targeted areas, and lead to more efficient intervention efforts.

## ACKNOWLEDGEMENTS

The authors thank the field staff at Vector and Vector‐Borne Diseases Research Institute, Tanzania. Permits were acquired from COSTECH and TAWIRI, Tanzania. This work was funded by the Biotechnology and Biological Sciences Research Council, the Department for International Development, The Economic and Social Science Research Council, The Natural Environment Research Council and the Defence, Science and Technology Laboratory, under the Zoonosis and Emerging and Livestock Systems (ZELS) programme (grant no. BB/L019035/1) and the UNICEF/UNDP/World Bank/WHO Special Programme for Research and Training in Tropical Diseases (TDR), the Canadian International Development Research Centre (IDRC), and by the European Union's Seventh Framework Program (FP7/2007‐2013) under grant agreement 221948, ICONZ (Integrated Control of Neglected Zoonoses). The funders had no role in study design, data collection and analysis, decision to publish or preparation of the manuscript.

## AUTHORS’ CONTRIBUTIONS

J.S.L., S.J.T., H.K.A. and L.J.M. jointly conceived the study. J.S.L., S.J.T., H.K.A., M.B. and F.M. were involved in field study design and data collection. J.S.L., S.J.T., M.C.S. and P.M.B. carried out data analysis. All authors contributed to writing and reviews of the manuscript. All authors gave final approval for publication.

## Data Availability

Data are available via Zenodo https://doi.org//10.5281/zenodo.1133429 (Lord et al., 2018).
