# Supplementary material for: Geostatistical models using remotely‐sensed data predict savanna tsetse decline across the interface between protected and unprotected areas in Serengeti, Tanzania
Source: J Appl Ecol. 2018 Feb 13;55(4):1997–2007. doi: 10.1111/1365-2664.13091 (PMC6032868; doi:10.1111/1365-2664.13091)

Fig. S1 Geostatistical model residuals against observed mean abundance for 2010 data >10 km inside the Serengeti National Park. a) *G. pallidipes*, b) *G. swynnertoni*

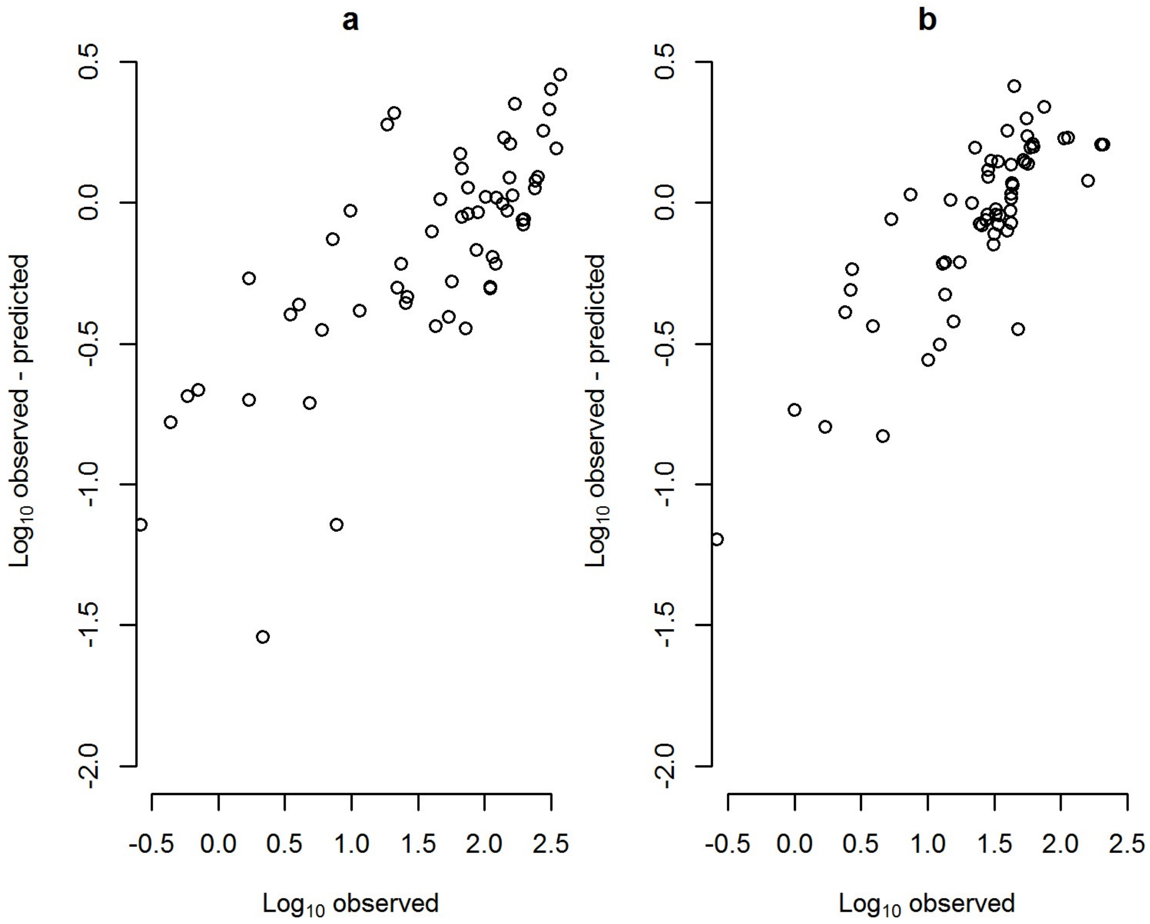

Supplement: Supplementary file 1 [file JPE-55-1997-s001.pdf]
