# Supplementary figures and images for: Geostatistical models using remotely‐sensed data predict savanna tsetse decline across the interface between protected and unprotected areas in Serengeti, Tanzania
Source: J Appl Ecol. 2018 Feb 13;55(4):1997–2007. doi: 10.1111/1365-2664.13091 (PMC6032868; doi:10.1111/1365-2664.13091)

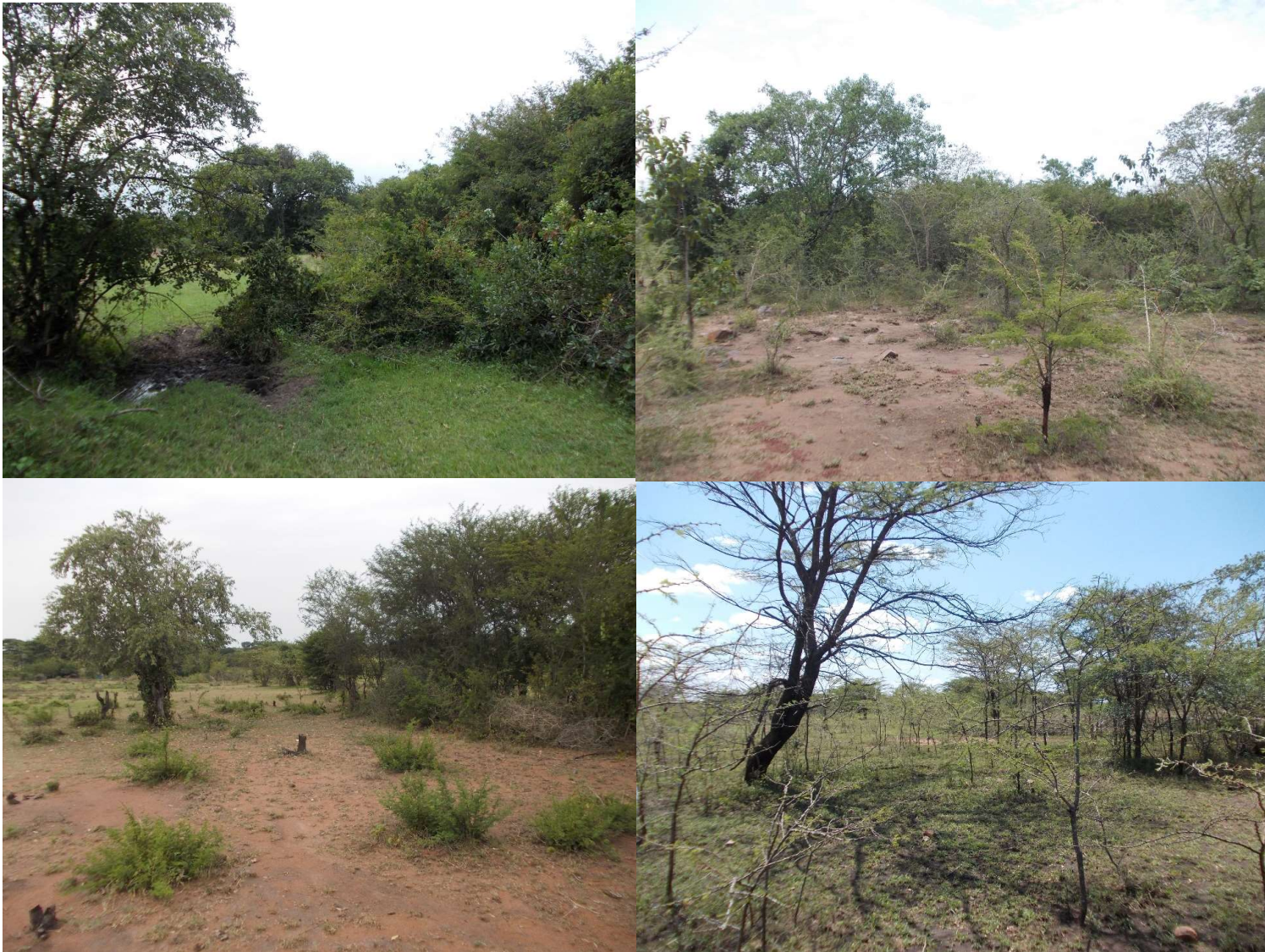

**Fig. S2 Field observations of locations outside protected areas, north of Grumeti Game Reserve.**

Supplement: Supplementary file 2 [file JPE-55-1997-s002.pdf]
