# Supplementary material for: Geostatistical models using remotely‐sensed data predict savanna tsetse decline across the interface between protected and unprotected areas in Serengeti, Tanzania
Source: J Appl Ecol. 2018 Feb 13;55(4):1997–2007. doi: 10.1111/1365-2664.13091 (PMC6032868; doi:10.1111/1365-2664.13091)
Supplement: Supplementary file 3 [file JPE-55-1997-s003.docx]

**Table S1. Mean values of remotely-sensed environmental variables between 2010 trap sites >10 km inside the Serengeti national Park (SNP) and 2015 trap sites across the interface between protected and unprotected areas.**

|  | **2010 sites >10 km inside SNP** | | **2015 sites across the interface between protected and unprotected areas** | |
| --- | --- | --- | --- | --- |
| **Variable** | **Mean** | **Standard deviation** | **Mean** | **Standard deviation** |
| Landsat 8 Band 7 | 0.12 | 0.02 | 0.13 | 0.03 |
| Normalised Difference Vegetation Index | 0.44 | 0.07 | 0.43 | 0.07 |
| Elevation (m) | 1405 | 67 | 1405 | 82 |
| Land surface temperature (^o^C) | 29.3 | 2.1 | 28.3 | 1.5 |
